# New Disulfide-Stabilized Fold Provides Sea Anemone Peptide to Exhibit Both Antimicrobial and TRPA1 Potentiating Properties

**DOI:** 10.3390/toxins9050154

**Published:** 2017-04-29

**Authors:** Yulia A. Logashina, Runar Gjerp Solstad, Konstantin S. Mineev, Yuliya V. Korolkova, Irina V. Mosharova, Igor A. Dyachenko, Victor A. Palikov, Yulia A. Palikova, Arkadii N. Murashev, Alexander S. Arseniev, Sergey A. Kozlov, Klara Stensvåg, Tor Haug, Yaroslav A. Andreev

**Affiliations:** 1 Shemyakin-Ovchinnikov Institute of Bioorganic Chemistry, Russian Academy of Sciences, ul. Miklukho-Maklaya 16/10, 117997 Moscow, Russia; yulia.logashina@gmail.com (Y.A.L.); mineev@nmr.ru (K.S.M.); july@ibch.ru (Y.V.K.); mosharova1988@mail.ru (I.V.M.); aars@nmr.ru (A.S.A.); serg@ibch.ru (S.A.K.); 2 Sechenov First Moscow State Medical University, Institute of Molecular Medicine,Trubetskaya str. 8, bld. 2, Moscow 119991, Russia; 3 Faculty of Biosciences, Fisheries and Economics, Norwegian College of Fishery Science, UiT—The Arctic University of Norway, NO 9037 Tromsø, Norway; runar.g.solstad@uit.no (R.G.S.); klara.stensvag@uit.no (K.S.); 4 Moscow Institute of Physics and Technology, Institutskyi per., 9, Dolgoprudnyi, 141700, Moscow, Russia; 5 Branch of the Shemyakin-Ovchinnikov Institute of Bioorganic Chemistry, Russian Academy of Sciences, 6 Nauki Avenue, 142290 Pushchino, Russia; dyachenkoigor7@gmail.com (I.A.D.); viktorpalikov@mail.ru (V.A.P.); palikowa2017@yandex.ru (Y.A.P.); murashev_an@mail.ru (A.N.M.); 6 Pushchino State Natural-Science Institute, 142290 Pushchino, Russian

**Keywords:** sea anemones, innate immunity, defensive strategies, TRPA1 receptor, antimicrobial

## Abstract

A novel bioactive peptide named τ-AnmTx Ueq 12-1 (short name Ueq 12-1) was isolated and characterized from the sea anemone *Urticina eques.* Ueq 12-1 is unique among the variety of known sea anemone peptides in terms of its primary and spatial structure. It consists of 45 amino acids including 10 cysteine residues with an unusual distribution and represents a new group of sea anemone peptides. The 3D structure of Ueq 12-1, determined by NMR spectroscopy, represents a new disulfide-stabilized fold partly similar to the defensin-like fold. Ueq 12-1 showed the dual activity of both a moderate antibacterial activity against Gram-positive bacteria and a potentiating activity on the transient receptor potential ankyrin 1 (TRPA1). Ueq 12-1 is a unique peptide potentiator of the TRPA1 receptor that produces analgesic and anti-inflammatory effects in vivo. The antinociceptive properties allow us to consider Ueq 12-1 as a potential analgesic drug lead with antibacterial properties.

## 1. Introduction

The phylum Cnidaria (including corals, hydroids, jellyfish, sea anemones and sea pens) is considered the oldest extant lineage of venomous animals, and species within this phylum are known to produce a variety of bioactive compounds that they utilize for capturing and killing prey and for defense [1]. All species within this phylum share a biological feature (which is likely due to common ancestry), the nematocysts; these are special cells designed to penetrate the skin of other animals and inject a venomous cocktail of compounds during hunting or defense [2,3,4]. Numerous bioactive peptides have been characterized from this phylum, including toxic and antimicrobial peptides, with molecular weights ranging from 3 to 15 kDa.

Antimicrobial peptides (AMPs) comprise a very diverse group of peptides containing both anionic and cationic, linear and cysteine-rich peptides [5]. Several AMPs have been characterized from cnidarians, including hydramacin-1 and c-arminin 1a, both from *Hydra magnipapillata* [6,7], periculin from *Hydra vulgaris* [8], aurelin from *Aurelia aurita* [9] and pd-AMP1, a 5.3 kDa peptide from *Phyllogorgia dilatata* [10]. Among sea anemones, *Anemonia sulcata* produces neurotoxin-2, which is both neurotoxic and proposed to be antimicrobial [11,12,13].

Sea anemones are well known producers of potent neuroactive peptides, acting on a diverse panel of ion channels, such as voltage-gated sodium (Na_V_) and potassium (K_V_) channels, transient receptor potential channels (TRP) and acid-sensitive ion channels (ASICs) [1,14,15,16,17].

The TRP superfamily of transmembrane non-selective ion-channels is involved in various perceptions including nociception and is therefore a target for analgesic treatment. All channels are believed to be tetrameric, consisting of subunits with six transmembrane domains [18,19]. Both antagonists and agonists of a number of these channels are promising drug candidates because antagonists can provide immediate pain relief while agonists can provide desensitization over time.

The transient receptor potential ankyrin 1 ion channel (TRPA1) was first cloned [20] and was identified as a receptor for noxious cold temperature [21]. Although controversy exists regarding its role as a thermosensor, its role in nociception is quite clear [22]. Agonists of TRPA1 activate sensory neurons in vivo causing acute pain, thermal and mechanical hyperalgesia and neurogenic inflammation. This is similar to the transient receptor potential vanilloid 1 (TRPV1), which is usually coexpressed with TRPA1 [23].

*Urticina* is a genus of sea anemones in the family Actiniidae. No antimicrobial peptides have previously been characterized in the sea anemone *Urticina eques*. However, a putative Type 1 neurotoxin (Ue-1) was discovered via RNA deep sequencing [24] and in the related species *U. grebelnyi*, a reversible inhibitor of both transient and sustained ASIC3 currents was characterized by Osmakov et al. [16]. Additionally, the genus has given rise to the 30 kDa cytolytic protein UcI and the phospholipase UcPLA2 of *U. crassicornis* [25,26], and the 28 kDa cardiac stimulatory and haemolytic protein UpI of *U. piscivora* [27].

Since sea anemones in general are known to be producers of biologically active peptides and owing to the fact that *U. eques* is largely unexplored, we aimed to isolate and identify new bioactive peptides in *U. eques*. We managed to isolate and characterize a novel peptide with an unusual cysteine distribution and a new molecular scaffold, providing both antimicrobial and antinociceptive properties.

## 2. Results

### 2.1. Isolation of Ueq 12-1

Specimens of the sea anemone *U. eques* were collected off the coast of Tromsø, Norway, and ectoderm secretions/mucus were obtained by electrical stimulation. The released exudate containing peptides and other hydrophobic components was desalted and concentrated using solid phase extraction (SPE). The antibacterial effect of the extract was observed against the Gram-positive strain *Corynebacterium glutamicum* at concentrations as low as 80 µg/mL. The same extract displayed an inhibiting effect on the TRPA1 ion channel at 0.1 mg/mL but did not affect the TRPV1 and TRPV3 channels in the Fluo-4-based intracellular calcium assay (data not shown).

**Figure 1:**
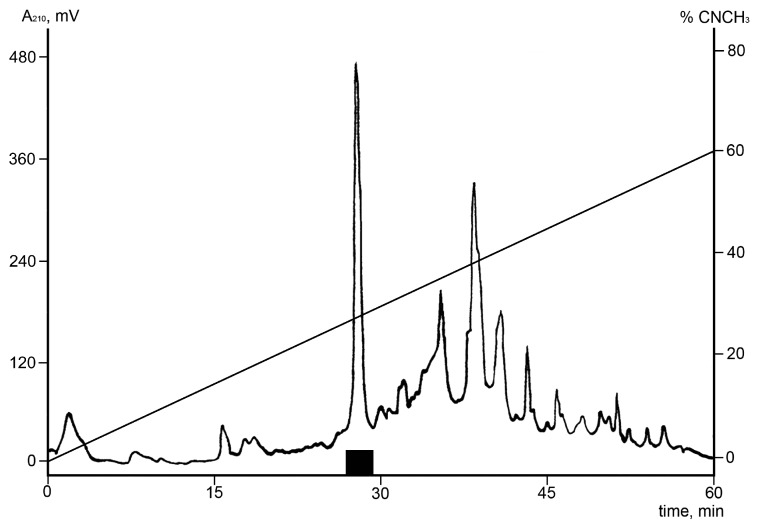
Isolation of Ueq 12-1 from *U. eques* mucus/secretions by reverse phase HPLC. A crude mucus extract (obtained after electrical stimulation and desalting) from *U. eques* was pre-purified by solid phase extraction and the eluate was subjected to RP-HPLC using a semi-preparative C_5_ column. Elution was performed with a linear gradient of 0–60% acetonitrile for 60 min at a flow rate of 5 mL/min. The fraction showing growth inhibitory activity against *C. glutamicum* and the ability to potentiate the TRPA1 ion channel and the peak containing Ueq 12-1 are shown with a bold black line (■).

In order to identify fractions with bioactive components, the extract was fractionated by preparative RP-HPLC (Figure 1). All the obtained fractions were subsequently tested for both antibacterial and TRPA1 activities. The fraction eluting after 27 min displayed antibacterial activity against *C. glutamicum.* In the Ca^2+^ influx assay, the same fraction was found to potentiate the TRPA1 ion channel (data not shown). The crude extract displayed a net inhibition of TRPA1, which is the opposite effect observed for the HPLC fraction: this is probably because of the presence of other interfering bioactive compounds.

The bioactive fraction was shown to consist of a peptide with a monoisotopic molecular mass of 4788.63 Da, as measured by HR-ESI-MS.

### 2.2. Ueq 12-1 Amino Acid Sequence Determination

Measurements of the molecular mass of the pyridylethylated peptide exceeded that of the native peptide by 1061.5 Da. This increase in mass is attributable to the addition of 10 pyridylethyl groups (10 × 106.14 Da) on 10 alkylated cysteine residues, which forms five disulfide bonds in the original peptide. A partial N-terminal sequence of Ueq 12-1 was successfully determined up to residue 34 by Edman degradation: CYPGQPGCGHCSRPNYCEGARCESGFHDCGSDHW, showing 6 of a total of 10 cysteines in the peptide.

**Figure 2:**
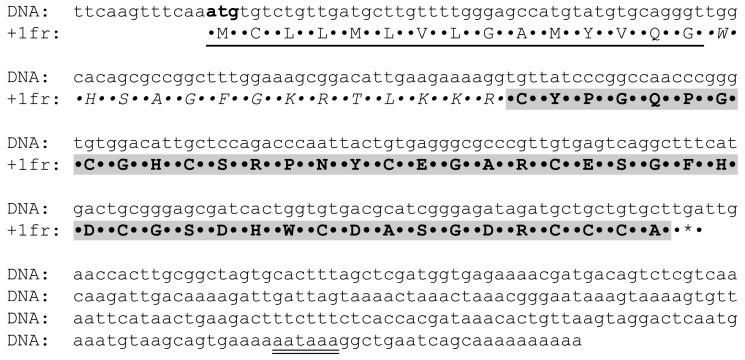
Complete cDNA sequence (ENA ID: LT600337) and the deduced amino acid sequence of the Ueq 12-1 precursor. The signal peptide sequence is underlined, the pro-peptide is shown in *italic*, and the mature Ueq 12-1 peptide sequence is marked in **bold**. The polyadenylation signal is $\underline{\underline{\mathrm{double}\;\mathrm{underlined}}}$ while an asterisk denotes the stop codon.

To elucidate the complete peptide sequence, degenerated primers were constructed on the basis of the partial primary peptide structure and were used to amplify the 3′-terminus of the transcript, using newly synthesized cDNA as a template. Using PCR with the universal primer T7Cap and degenerated primers, a fragment of about 400 bp was obtained. The PCR product was cloned into a pAL-TA vector and sequenced. Based on the nucleotide sequence of the found gene, reverse primers UE-r1 and UE-r2 were synthesized for 5′-RACE. A DNA fragment of about 300 bp was obtained after PCR and subsequently cloned into a pAL-TA vector and sequenced. The cDNA encoding the precursor (ENA ID: LT600337) and the deduced peptide sequence of Ueq 12-1 is shown in Figure 2. The complete deduced peptide sequence of Ueq 12-1 contains 74 amino acids. Comparison of the partial amino acid sequence found by N-terminal sequencing and the peptide sequence deduced from cDNA revealed that the precursor contains a pre-region of 29 amino acid residues upstream of the mature Ueq 12-1 sequence. Analysis by SignalP 4.1 indicated that a cleavage site of a signal peptide was located between amino acid Gly15 and Trp16 in the sequence. Thus, the precursor peptide contains a signal peptide of 15 amino acids and a propeptide sequence between Trp16 and Arg29 (14 amino acids) followed by the mature peptide consisting of 45 amino acids. Assuming disulfide bonds between the 10 cysteine residues the deduced peptide has a calculated average mass of 4792.13 and a monoisotopic mass of 4788.62 Da. This mass is nearly identical to the monoisotopic mass of the purified peptide as determined by HR ESI mass spectrometry (4788.63 Da). The mature peptide is anionic with a net charge of −3 at neutral pH, and a calculated pI of 5.28.

### 2.3. Production of Recombinant Ueq 12-1

To obtain larger quantities of the peptide for bioactivity screening and structure-function investigations, an *Escherichia coli* expression system was utilized. Ueq 12-1 was expressed as a fusion protein with thioredoxin (Trx), which promotes the folding of cysteine-rich peptides into their native conformation [28]. A synthetic gene product encoding Ueq 12-1 was constructed from synthetic oligonucleotides by PCR and was cloned into the expression vector pET32b(+). The *E. coli* SHuffle cells were transformed by the resulting construction pET32b(+)-Ueq 12-1. The fusion protein (Trx-Ueq 12-1) was isolated using metal affinity chromatography, and was subsequently subjected to CNBr cleavage and purification by reverse-phase HPLC to obtain the pure recombinant peptide. The recombinant Ueq 12-1 had the same retention time as the native peptide when fractionated by reverse-phase HPLC (data not shown), producing the same effect in electrophysiological tests on TRPA1 and in behavioral tests in mice. This implies that they were identical. Moreover, analysis of 1H NMR spectra revealed the complete identity of natural and recombinant Ueq12-1 samples at pH 3.0. The total yield of Ueq 12-1 was approximately 0.7 mg per liter of bacterial culture. The average molecular mass of the recombinant product, determined by MALDI mass spectrometry (4792.60 Da), was in accordance with the theoretical mass calculated from the sequence data (4792.13 Da), and the average mass of the native peptide measured by HR ESI mass spectrometry (4792.45 Da).

### 2.4. Spatial Structure of Ueq 12-1 in Water Solution

**Figure 3:**
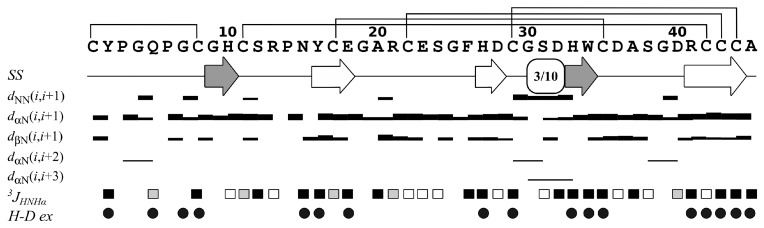
Overview of the NMR data, defining the secondary structure of Ueq 12-1. Hydrogen-deuterium exchange rates of HN protons with solvent (H-D ex), ^3^J_HNHα_ couplings, secondary structure of Ueq 12-1 (SS) and NOE connectivities (d_ij_) are shown versus the protein sequence. Widths of the bars correspond to the relative intensity of cross-peak in the 80 ms NOESY spectrum. Circles denote H_N_ protons, which exchange with solvent with characteristic times longer than 15 min. Black, gray and white rectangles denote residues with large (>8 Hz), medium (6–8 Hz) or small (<6 Hz) magnitudes of ^3^J_HNHα_, respectively. Elements of secondary structure are shown on the separate line, β-strands are indicated by arrows, color of arrows indicates the involvement of strand in parallel (grey) or antiparallel (white) β-sheet.

In order to determine the fold of Ueq 12-1, the structure of the protein was resolved in water solution by NMR spectroscopy. A set of 20 possible structures was calculated using the CYANA program [29] from 100 random starting points using the following experimental data: upper and lower NOE-based distance restraints, torsion angle restraints, and hydrogen bonds restraints (Figure 3). The statistics of the obtained set of Ueq 12-1 structures are shown in Table 1.

The set of structures is characterized by a relatively low CYANA target function and residual restraint violations, and a low RMSD value for backbone atoms, suggesting that the structure of Ueq 12-1 is well-converged and defined accurately by the experimental data.

**Figure 4:**
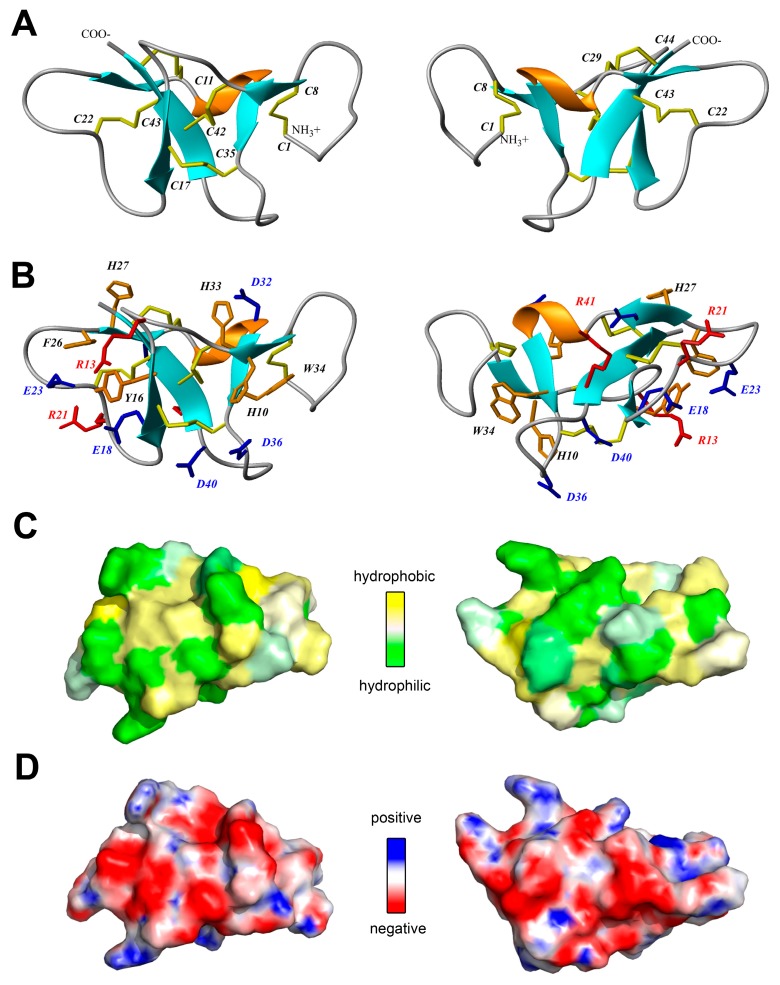
Two-sided view of the determined spatial structure of Ueq 12-1 (**PDB ID: 5LAH**). (**A**,**B**) Ribbon representation. Disulfide bridges, positively and negatively charged side chains and aromatic residues are shown by yellow, red, blue and orange sticks, respectively; (**C**) Hydrophobicity of the Ueq 12-1 surface. Contact surface of Ueq 12-1 is colored from yellow (hydrophobic) to green (hydrophilic), according to the molecular hydrophobic potential (MHP) [30]; (**D**) molecular surface of Ueq 12-1 is painted according to the electrostatic potential.

According to the NMR data, Ueq 12-1 is organized in a peculiar W-shaped structure (Figure 4A,B). The core of the structure is formed by a 3-strand antiparallel β-sheet (strands Y16–E18, H27–D28 and R41–C44), a small 2-strand parallel β-sheet (G9–H10, H33–W34) and one turn of a 3–10 helix (G30–D32). The obtained structure consists of three large loops including 8 β-turns of various types (Y2–Q5, IV; Q5–C8, IV; S12–N15, VIa1; R13–Y16, IV; E18–R21, IV; E23–F26, IV; C35–S38, VIII; A37–D40, IV) and it is stabilized by 5 disulfide bridges (C1–C8, C11–C42, C17–C35, C22–C43 and C29–C44). Analysis of the hydrogen-deuterium exchange rates of the protein amide groups revealed 19 hydrogen bonds. Besides, a number of aromatic and negatively charged residues in one of the loops of Ueq 12-1 that are involved in stacking and π-anion interactions are observed (Y16, F26, E18, E23). The surface of the peptide is polar without pronounced clusters of positively or negatively charged side chains (Figure 4C,D).

### 2.5. In Vitro Antimicrobial Activities of Ueq 12-1

**Figure 5:**
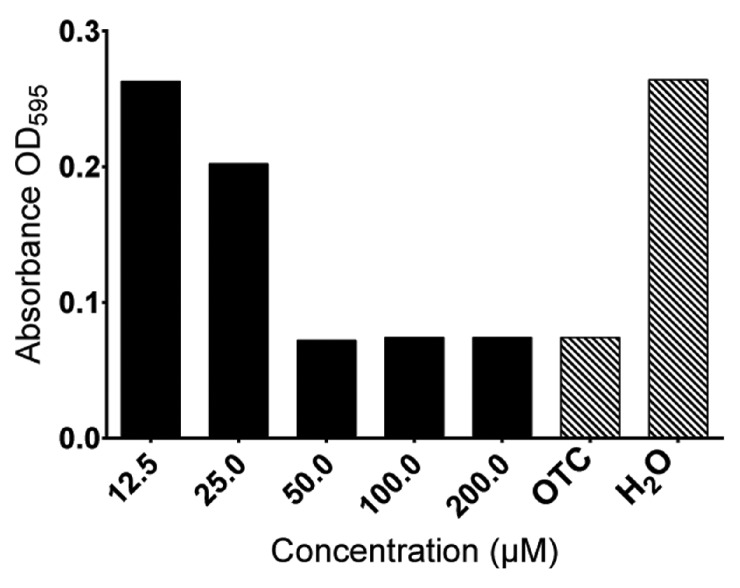
Antibacterial activity toward *C. glutamicum* of Ueq 12-1 in concentrations ranging from 12.5 to 200 µM. The peptide is antibacterial in concentrations ≥50 µM. OTC (tetracycline) (40 µM) and H_2_O served as controls.

Ueq 12-1 was purified in its natural form and its antimicrobial potential was estimated against selected bacterial strains. The test bacteria were represented by two Gram-negative strains *(E. coli* and *P. aeruginosa*) and two Gram-positive strains (*S. aureus* and *C. glutamicum*). The three strains (*E. coli, P. aeruginosa* and *S. aureus*) are all relevant laboratory strains, similar to disease-causing bacterial strains in humans. *C. glutamicum* was chosen because of high sensitivity to antimicrobial compounds which increases the possibility of detecting minimal amounts of antimicrobial peptides [31]. As far as we know, none of these bacterial strains cause disease in sea anemones. Therefore, they have limited ecological relevance. The peptide had moderate activity against *C. glutamicum* (MIC = 50 µM), but showed minor activity against *S. aureus* and no activity against *P. aeruginosa* and *E. coli* (MIC > 200 µM) (Figure 5).

### 2.6. Effect of Ueq 12-1 on TRPA1 Activity

**Figure 6:**
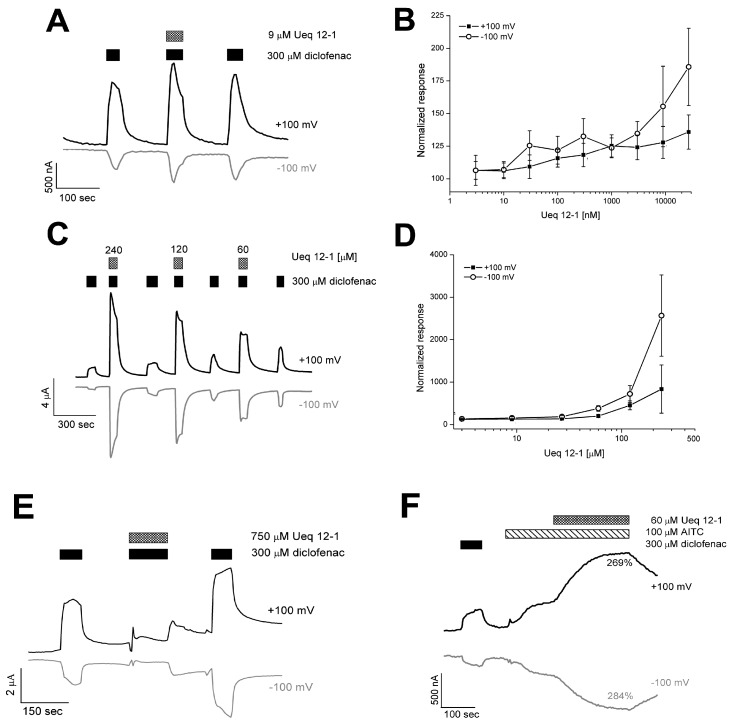
TRPA1 currents evoked in response to exposure to diclofenac (300 μM) and AITC (100 μM) in the presence of Ueq 12-1. (**A**) representation trace for potentiating effect of Ueq 12-1 at a concentration of 9 μM on diclofenac-induced currents; (**B**) data on potentiating activity of peptide at a concentration of 3–30 μM on diclofenac-induced currents (shown as mean ± SD, *n* = 5–7); (**C**) representation trace for potentiating effect of Ueq 12-1 at a concentration of 60–240 μM; (**D**) data on potentiating activity of peptide at a concentration of 60–240 μM on diclofenac-induced currents (shown as mean ± SD, *n* = 5–7); (**E**) representation trace for defunctionalization of TRPA1 in the presence of Ueq 12-1 at a concentration of 750 μM; (**F**) representation trace for potentiating effect of Ueq 12-1 at a concentration of 60 μM on AITC-induced current.

Ueq 12-1 was found to potentiate the allyl isothiocyanate (AITC) induced TRPA1 Ca^2+^ influx in a stably transfected Chinese Hamster Ovary (CHO) cell line up to 35 ± 10% at 1 µM. The electrophysiological effect of Ueq 12-1 was tested on TRPA1 expressed in *Xenopus laevis* oocytes. At 240 µM, Ueq 12-1 did not cause any current in the TRPA1-expressing oocytes, but it significantly potentiated the activation of TRPA1 induced by AITC or diclofenac. Dose-response analysis of potentiation was carried out using oocytes activated by 300 µM diclofenac (Figure 6). At concentrations ranging from 0.3 to 27 µM, Ueq 12-1 moderately potentiated TRPA1 outward currents up to 120–135% and inward currents up to 132–186% (Figure 6A,B). Higher concentration of the peptide caused significantly more potentiation of the TRPA1 currents (at 60 µM − 200 ± 23% (outward) and 378 ± 65% (inward); at 120 µM − 453 ± 104%(outward) and 719 ± 98% (inward), data shown as mean ± SD) (Figure 6C,D). Application of Ueq 12-1 at a concentration of 240 µM (together with diclofenac) dramatically increased the TRPA1 outward and inward currents up to 835 ± 566% and 2559 ± 959%, respectively (Figure 6C,D). At concentrations above 750 µM, Ueq 12-1 caused no standard response to agonist, probably due to defunctionalization of the receptor in the presence of peptide (Figure 6E). The same potentiating effect of peptide was observed for AITC-induced currents of TRPA1 (Figure 6F). Ueq 12-1 at a concentration of 60 µM produced neither agonistic nor antagonistic activity on rat TRPV1 and human TRPV3 receptors in fluorescent assay of calcium influx in CHO cell lines stably expressing these receptors.

### 2.7. In Vivo Bioactivites of Ueq 12-1

**Figure 7:**
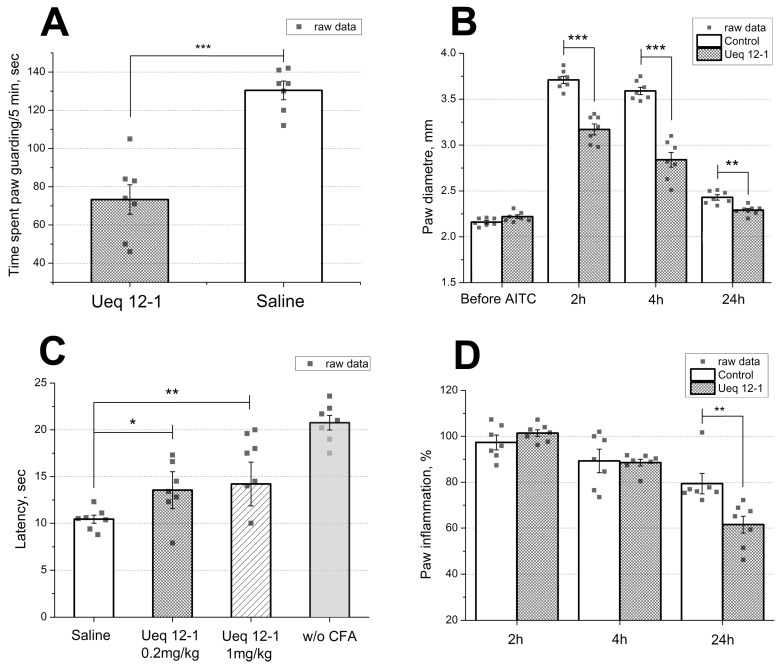
Efficacy of the peptide Ueq 12-1 in animal models. (**A**) Ueq 12-1 (0.2 mg/kg, i.v.) significantly reduce behavioral response and (**B**) inflammatory response to injection of AITC; (**C**) effect of Ueq 12-1 on the mice withdrawal latency on a hot plate in the thermal hyperalgesia test after CFA injection and (**D**) on CFA-induced paw inflammation (0.2 mg/kg). Results presented as mean ± SEM; ***, *p* < 0.001; **, *p* < 0.01; *, *p* < 0.05; versus saline group (ANOVA followed by a Tukey’s test); *n* = 7 for all experiments.

**Figure 8:**
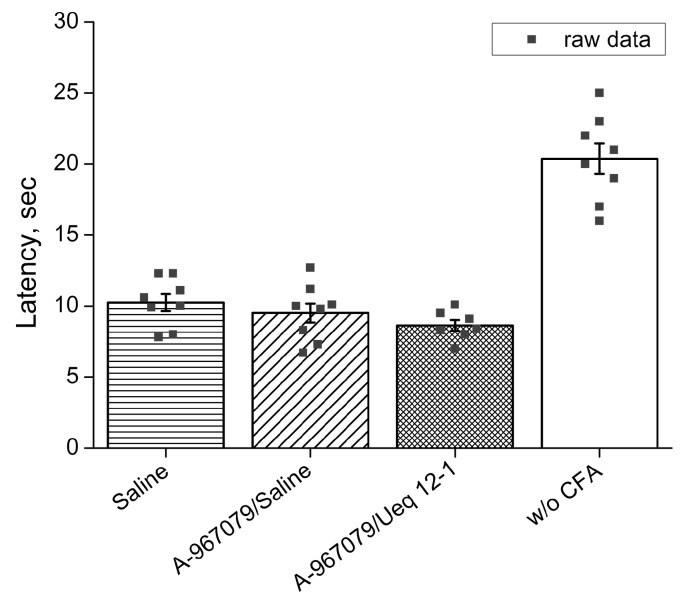
Selective TRPA1 antagonist A-967079 (20 mg/kg, p.o.) completely blocked the effect of peptide Ueq 12-1 (0.2 mg/kg, i.v.) on thermal hyperalgesia (*n* = 7–8).

Injection of Ueq 12-1 (10 μL, 50 μM solution) into the mouse hind paw did not cause pain, paw edema or significant thermal hyperalgesia during the 2 h experiment. The TRPA1 agonist AITC was injected into the mice to provoke pain behavior associated with TRPA1 activation. The dosage of AITC was chosen to induce half-maximal effect (20 µL, 0.5% in saline). Pretreatment of mice by intravenous injection of Ueq 12-1 (0.2 mg/kg) significantly reduced AITC-induced nocifensive behavior. Paw licking and time spent guarding were significantly decreased (49% and 43% reduction, respectively) in the Ueq 12-1 treated mice (Figure 7A). Moreover, paw edema caused by AITC was significantly reduced by 39 and 57% 2 and 4 h after injection, respectively (Figure 7B). Additionally, the influence of intravenous injection of Ueq 12-1 (0.2 mg/kg) on non-specific inflammation was tested using the Complete Freund’s Adjuvant (CFA) test. Inflammation was induced by injection of CFA into the hind paw and Ueq 12-1 (0.2 mg/kg) was administrated 24 h afterward. Ueq 12-1 reversed thermal hyperalgesia within 30 min after i.v. administration (Figure 7C) and decreased paw edema by 27% 24 h after injection (Figure 7D). Pre-treatment of mice by selective TRPA1 antagonist A-967079 (20 mg/kg, p.o. 1 h before peptide) completely blocked the effect of peptide Ueq 12-1 (0.2 mg/kg, i.v.) on CFA-induced inflammation (Figure 8).

The response to the peptide observed in the behavioral tests was not a result of sedation or behavior impairment because administration of Ueq 12-1 did not change the normal behavior of mice in the open field test (results not shown).

## 3. Discussion

Sea anemones are known to produce an array of biologically active peptides of different structures and folds, and many of them are promising for pharmaceutical drug development [1]. Sea anemone toxins comprise mainly proteins and peptides that are cytolytic or neurotoxic, with their potency varying with the structure, target molecule and mechanism of action. Sea anemone toxins include voltage-gated Na^+^ and K^+^ channels toxins, acid-sensing ion channels (ASIC) toxins, cytolysins, Kunitz-type protease inhibitors and toxins with Phospholipase A2 activity [1,32]. In the present study, we have isolated and characterized a novel peptide Ueq 12-1 with a unique disulfide fold that possesses both antimicrobial and antinociceptive properties.

### 3.1. Primary Structure Analysis of Ueq 12-1

Ueq 12-1 is a 45-amino acid anionic peptide containing 10 cysteines engaged in five intramolecular disulfide bonds. No significantly homologous peptides were found in the databases. Based on earlier results, sea anemones may be considered leaders among the venomous animals in terms of the structural variety of polypeptides in their venoms and mucus. Polypeptides from the animal venom usually contain an even number of cysteine residues normally involved in the formation of disulfide bonds forming a rigid structure, more resistant to proteolysis [17,33].

Ueq 12-1 displays no sequence homology to known characterized sea anemone neurotoxins. The majority of characterized cysteine-rich peptides from sea anemones consist of 25–60 amino acid residues, 4–10 cysteine residues and a considerable number of positively charged residues (Lys, Arg) forming positively charged clusters on the surface [1,34]. Almost 50% of the reported peptides are ligands of sodium or potassium channels, and positively charged and hydrophobic residues play a leading role in toxin-channel interaction.

A classification of SWISS PROT annotated cysteine-rich sea anemone peptides by their cysteine distribution has recently been developed [32]. All the annotated peptides are classified into 11 families with characteristic cysteine patterns that suggest 11 variants of polypeptide folding. Furthermore, at least 16 different motifs of cysteine distribution in the polypeptides have been identified [32]. The peptide Ueq 12-1 has a unique cysteine pattern and therefore it cannot be assigned to any of the 11 distinct structural subfamilies of the known sea anemone peptides. Ueq 12-1 thus represents a new family of peptides with the motif C6C2C5C4C6C5C6CCC# (where # is 1 − 9 aa). The given full name is τ-AnmTx Ueq 12-1 (short name Ueq 12-1). This means that the peptide is the first representative of a 12th subgroup according to the classification proposed by Mikov [32].

No highly homologous peptides or gene products were found using the NCBI BLAST search engine in non-redundant protein sequences (nr) database and Transcriptome shotgun assembly proteins (Tsa_nr) database or within searchable antimicrobial peptide databases. Even the StellaBase (the *Nematostella vectensis* genomics database), Pocillopora Transcriptomics Database (*Pocillopora damicornis*, cauliflower coral) and EdwardsiellaBase (the *Edwardsiella lineata* Genomic Database) returned no homologous sequences. Furthermore, no conservative protein domains were detected using the InterProScan tool.

We found some similarities in the cysteine distribution pattern with small cysteine-rich peptides (SCRiPs) from corals and sea anemones extracted from EST databases [35]. Additionally, one cDNA coding for SCRiP peptide (TSA: *Anthopleura elegantissima* comp63456_c0_seq1 transcribed RNA sequence, GBYC01024820.1) similar to Ueq 12-1 was found in Transcriptome shotgun assembly (TSA) database using TBLASTN search. We should note that a variety of different cDNAs coding for SCRiPs peptides could be identified in the TSA database using known SCRiPs as a query and TBLASTN search in Cnidarians transcriptomes but the homology of SCRiPs from different species is very low.

**Figure 9:**
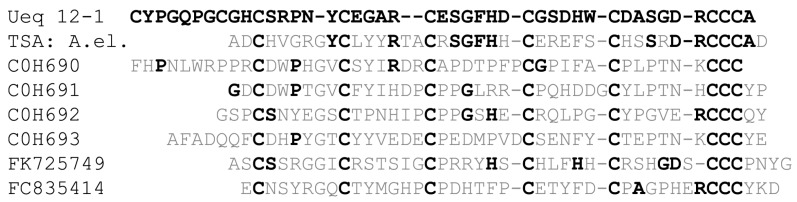
Sequence alignment of Ueq 12-1 (**UniProt Protein Database ID: C0HK26**) and small cysteine rich peptides (SCRiPs). TSA: A. el. − peptide obtained from TSA: *Anthopleura elegantissima* comp63456_c0_seq1 transcribed RNA sequence, GBYC01024820.1 by translation. C0H690 *Acropora millepora* (SCRiP1); C0H691 *A. millepora* (SCRiP2); C0H692 *A. millepora* (SCRiP3); C0H693 *Montipora capitata* (SCRiP1a); FK725749 *Anemonia viridis*; and FC835414 *Metridium senile*. The residues identical to Ueq 12-1 are highlighted in bold.

An identifying feature for both Ueq 12-1 and SCRiPs is a triplet of cysteines located in the C-terminus (Figure 9). Since all SCRiPs sequences are deduced from cDNA, no data is available regarding their function and spatial structure. SCRiPs contain only eight cysteine residues, and their distribution is similar to the eight C-terminal cysteines of Ueq 12-1 (Figure 9). Apart from the cysteine-motif, there is no significant primary structure homology between Ueq 12-1 and SCRiPs. Most probably, Ueq 12-1 and SCRiPs reflect the evolution of an ancient venom component and a common evolutionary origin of cnidarian toxins [35].

### 3.2. Spatial Structure Analysis of Ueq 12-1

**Figure 10:**
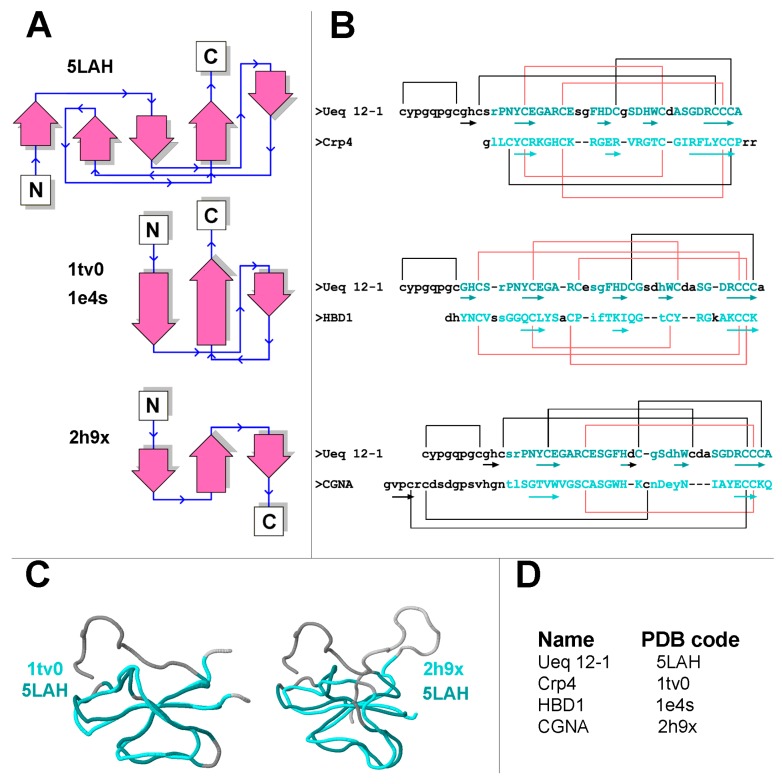
Comparison of Ueq 12-1 with other defensin like-peptides. (**A**) Schematic representation of the secondary structures of Ueq 12-1 and defensin/defensin-like peptides; (**B**) The primary structure alignment is based on spatial structure homology with the mouse alpha-defensin 4 (1tv0), HBD1 (1e4s) and CGNA toxin (2h9x). The amino acid sequences are aligned according to equivalent topological structure as determined by the PDBeFold algorithm. Beta-strands are shown with arrows. Residues that entered spatial alignment are shown in shades of cyan, whereas residues with distance between Cα atoms more than 3 Å are shown in small letters. Similar disulfide bonds are shown in red; (**C**) Backbone spatial alignments for Ueq 12-1 and defensin-like peptides − mouse alpha-defensin 4 Crp4 (1tv0) and CGNA toxin (2h9x) (**D**) Names and PDB codes of peptides.

Analysis of the spatial structure of Ueq 12-1 revealed significant similarity to some defensin-like peptides of mammals, platypus and some sea anemone toxins of the disulfide-rich, nearly all-β structural defensin-like fold (SCOP family g.9.1.1) [36]. Analysis of the 3D structure homology using the PDBeFold analysis tool [37] revealed that Ueq 12-1 could be aligned with the spatial structures of several peptides (Table 2 and Figure 10). These were the mouse alpha-defensin 4 (Cryptdin-4, 1tv0), the defensin-like peptide from platypus venom DLP-2(4) (1zue, 1zuf), the human beta-defensins HBD-2 and HBD-1 (1e4q, 1e4s), the plant cyclotyde Kalata-B12 (2kvx), and two peptide toxins derived from sea anemones: Anthopleurin-A (1ahl) and CGNA toxin (2h9x). Thus, despite the absence of homology in the primary structure, the backbone of peptide Ueq 12-1 forms a similar to defensin-like peptides fold (Figure 10, Table 2). It is also noteworthy that Ueq 12-1 is less similar to sea anemone defensin-like peptides compared to mammalian defensins. The significance of structural alignment was evaluated by the Q-score that represents the quality function of Cα-alignment, maximized by the Secondary Structure Matching (SSM) alignment algorithm. This is a complex characteristic, which takes into account both the alignment length and the RMSD (Root Mean Square Deviation, calculated between the Cα-atoms of the matched residues in the best 3D superposition of the query and target structures).

It is important to note that the Q-score was rather low (0.46–0.38) for the peptides that have a defensin-structure domain such as the mouse alpha-defensin 4 (1tv0), DLP-2(4) (1zue, 1zuf) and hBD2 (1e4q), and it was very low (~0.25) for the sea anemone defensin-like peptides anthopleurin-A (1ahl) and CGNA toxin (2h9x). For comparison, the Q-score for the structural alignment of anthopleurin-A (1ahl) and CGNA toxin (2h9x) was 0.7; for DLP-4 (1zuf) and hBD2 (1e4q) it was 0.52; and for alpha-defensin 4 (1tv0) and beta-defensin hBD2 (1e4q) it was 0.47.

All the defensin/defensin-like peptides described above contain 6 cysteines in their primary structure that form 3 disulfide bridges, whereas Ueq 12-1 contain 10 cysteines and 5 disulfide bridges. Furthermore, none of these peptides contain the CCC motif in the C-terminus found in Ueq 12-1. There are two types of defensins differing in disulfide bond configuration. In α-defensins (1tv0), the three disulfide bridges have the topology C1–C6, C2–C4, C3–C5, whereas the disulfide arrangement of β-defensins (1e4q, 1zue, 1e4s, 1ahl, etc.) is C1–C5, C2–C4, C3–C6 [38] (Figure 10). In Ueq 12-1, the cysteine residues are paired in a C1–C2, C3–C8, C4–C7, C5–C9, C6–C10 configuration. The disulfide bridges C3–C8, C4–C7, C5–C9 of Ueq 12-1 correspond to the β-defensin linkage, whereas the C1–C2 and C6–C10 pairings are additional bonds, which is characteristic for this peptide (Figure 10B). The disulfide bond C1–C2 stabilizes the unique N-terminal loop of Ueq 12-1.

A defensin-like structural fold is shown for the sea anemone neurotoxins and is referred to as structural group 1. These peptides affect a variety of cellular targets such as sodium, potassium and acid-sensing channels. The closest structural homologues of Ueq 12-1 isolated from sea anemones are sodium channels toxins rather similar in primary structure (~50% of identity): Anthopleurin-A (1ahl) from *Anthopleura xanthogrammica* [39] and CGNA toxin (2h9x) from *Condylactis gigantea* [40]. Both are referred to as group 1a toxins [32]. Anthopleurin-A is a 49-residue polypeptide that produces delayed inactivation of the myocardial voltage gated Na^+^ channel [41]. The CGNA toxin contains 47 amino acid residues and act on tetrodotoxin-sensitive voltage-gated sodium channels, increasing the inactivation time constant and the rate of recovery from inactivation [40]. Both toxins contain 6 cysteines, but only one (C3–C6) of the three disulfide bridges is similar to Ueq 12-1 in the structural alignment (Figure 10B).

Figure 10A is also a schematic representation of the secondary structural elements of Ueq 12-1 and defensin-like peptides. A considerable part of the Ueq 12-1 molecule is arranged very similarly to the defensin-like fold that is also found in some bioactive peptides derived from sea anemones and platypus. All the known mammalian defensins adopt a similar overall structure, i.e., a triple-stranded antiparallel β-sheet [38]. The core of the Ueq 12-1 structure is also formed by a 3-strand antiparallel β-sheet (strands Y16–E18, H27–D28 and R41–C44). Significant dissimilarities of the Ueq 12-1 structure from the defensin-like peptides are the additional small 2-strand parallel β-sheet (G9–H10, H33–W34) and one turn of a 3–10 helix (G30-D32) (Figure 3 and Figure 9). Moreover, Ueq 12-1 has an unusual cysteine distribution similar to previously reported SCRiPs. Nothing is known about the SCRiPs spatial structure but it has been shown that some of them could be toxic against *Danio rerio* at rather high concentrations (230 µg/mL) [35]. Based on our data on the spatial structure of Ueq 12-1, SCRiPs most likely also have a defensin-like fold, as their cysteine distribution is similar to the defensin-like domain of Ueq 12-1. Some of the SCRiPs could therefore also have antimicrobial properties and target neuronal channels.

### 3.3. Bioactivity Analysis of Ueq 12-1

Ueq 12-1 is one of the main components found in *U. eques* ectoderm mucus/secretions. The peptide seems to display different defense and offense functions because it inhibits bacterial cell growth and potentiates the TRPA1 ion channel in vitro. Antibacterial activity can be useful for decreasing the probability of infection after damage in vivo, and the effect on TRP channels could mislead prey, predators or competitive species on the sea floor. TRPA1-like proteins are very conserved, take part in mechanosensitivity and response to external stimuli [42]. For instance, TRPA1-like ion channels in sea anemones seem to be responsible for vibration-dependent discharge of nematocysts [43].

The two different biological activities of Ueq 12-1 were discovered independently. Ueq 12-1 significantly inhibited growth of the Gram-positive *C. glutamicum* at 50 µM. This effect could be associated with the partial defensin-like structure found in Ueq 12-1, as defensins are known to have antibacterial activities [38].

The capability of defensins to interact with microbial membranes is determined by the shape of the molecules, the ratio of polar to hydrophobic residues on the peptide surface, and the total positive charge given through basic amino acids rather than by their individual primary structures [44]. Usually, increasing the overall positive charge will increase the antimicrobial activity of AMPs due to the selective action of AMPs on negatively charged bacterial membranes. Indeed, the AMP cryptidin-4 (Crp4, charge +8) more effectively associates with negatively charged biological membranes than neutral membranes [45]. The antimicrobial activity of Ueq 12-1 is most probably associated with other structural elements, as the peptide is negatively charged at neutral pH (pI = 5.28) but still maintains antimicrobial activity. Meanwhile, the triple-stranded β-sheet with its specific arrangement of disulfide bridges in defensins only provides stability to their structures, but is not an obligate determinant of antimicrobial activity [44].

TRPA1 is a nonselective cation channel that is mainly expressed in sensory neurons and is activated by exogenous electrophilic compounds (and a few other chemicals) as well as endogenous ligands produced during inflammation or oxidative stress [46]. TRPA1 activation in the sensory neurons produce both signals of pain or itching to the CNS and the release of pro-inflammatory peptides provoking local inflammatory responses [42,46]. The TRPA1 receptor is therefore considered a potential target for novel anti-inflammatory and analgesic drugs. In this study, Ueq 12-1 significantly potentiated the response of TRPA1 to AITC and diclofenac in Ca^2+^ imaging and electrophysiological experiments. However, we could not obtain a classical dose-response curve for the potentiating effect because the peptide at high concentrations completely blocked the response of the receptor. This is an unusual result, as the only known peptide potentiating TRPA1, Ms 9a-1 from sea anemone Metridium senile, had the standard concentration-depended effect. [47].

Ueq 12-1 was found to be a bifunctional molecule displaying both antimicrobial and TRPA1 receptor potentiating activity (Figure 5 and Figure 6). Multifunctionality seems to be a common feature of sea anemone peptides. For instance, they may combine serine protease inhibitor properties with the inhibition of voltage-gated potassium channels [48,49] or the TRPV1 receptor [17,50]. The defensin-like peptide neurotoxin 2 (ATX-II) isolated from the sea anemone *Anemonia sulcata* was reported to affect sodium channels gating and have antimicrobial properties [11]**.** This multifunctionality can be an optimal strategy for the construction of biologically active peptides allowing sea anemones to be active predators through the production of neurotoxins and resist bacterial infections caused by the eventual injury of the tentacles. It is noteworthy that human β-defensin 2 (Hbd-2), the structural homologue of Ueq 12-1, possess antimicrobial properties, act as an opener of large-conductance Ca^2+^-activated potassium (BKCa)-mSlo+hβ1 channels and inhibit Kv1.3-channels [38,51,52]. These studies highlight the multifunctionality observed for this particular spatial fold.

Injection of Ueq 12-1 into mice did not cause pain or hyperalgesia. Ueq 12-1 produced a significant reduction of pain response to AITC, a TRPA1 agonist, and reduced CFA-induced hyperalgesia and inflammation (Figure 7). Most probably, this peptide can potentiate and defunctionalize the TRPA1 receptor on sensory neurons or desensitize neurons. Inhibition of the TRPA1 function in vivo by antagonist A-967079 eliminated the effect of Ueq 12-1 on thermal hyperalgesia (Figure 8). Therefore, activation of TRPA1 is crucial for Ueq 12-1 analgesic effect of Ueq 12-1. In our study, defunctionalization of the TRPA1 receptor was achieved only at extremely high concentrations of the peptide, but in mammals, a great number of endogenous TRPA1 modulators can affect receptor activation [42,46] and change sensitivity to the peptide. Additionally or alternatively, Ueq 12-1 can potentiate the activation of TRPA1 to low concentrations of endogenous agonists, producing weak but sustained activation of the receptor that leads to desensitization of TRPA1-expressing neurons. Such an effect of weak activation of TRPA1 on the function of neurons has been shown for parthenolide, a partial agonist of TRPA1, isolated from the flower feverfew (*Tanacetum parthenium*) [53]. Parthenolide is considered the main component of feverfew that is responsible for the anti-migraine effect of this herbal remedy [53]. Therefore, potentiation/desensitization of TRPA1 ion channels might be an efficient treatment of pain and inflammation.

## 4. Conclusions

We should conclude that τ-AnmTx Ueq 12-1 is a unique, natural peptide possessing a new spatial structure fold, exhibiting an antimicrobial activity, potentiating TRPA1, and producing an analgesic effect in animal models of pain. Therefore, Ueq 12-1 could be considered as potential analgesic drug lead with antibacterial properties.

## 5. Materials and Methods

### 5.1. Animal Material and Sample Preparation

Live specimens of *Urticina eques* (Gosse, 1858) were collected by divers off the coast of Tromsø, Norway, during the summer/autumn of 2012. The animals were placed in tubs with continuously flowing, unaltered seawater (4–8 °C), from one to eight weeks before sampling.

The approach for electrical stimulation is inspired by the work of [54] where antimicrobial peptides were harvested from skin/mucus secretions of frogs. Ectoderm secretions were collected by removing the specimens from the tubs and subjecting them to electrical stimulation with a constant current of 300 mA, a non-fixed voltage of 10 Hz, and an impulse duration of 10 ms. The released exudate was rinsed of the animals with a 10 mM ethylenediaminetetraacetic acid (EDTA)/0.1 mM phenylmethylsulfonyl fluoride (PMSF) solution and centrifuged.

Salt was removed from the aqueous supernatant by reversed phase solid phase extraction (RP-SPE) using a Supelco visiprep vacuum manifold (Sigma-Aldrich, St. Louis, MO, USA) with a C_18_ 35cc Sep-Pak cartridge (Waters, Milford, MA, USA). The cartridge was conditioned with acetonitrile (MeCN)/0.05% trifluoroacetic acid (TFA) and equilibrated with 0.05% TFA. After sample loading, the cartridge was washed with 10% MeCN/0.05% TFA, and elution of the retained material was carried out with 80% MeCN/0.05% TFA. The eluate was lyophilized and kept frozen at −20 °C until further analysis.

### 5.2. High Performance Liquid Chromatography (HPLC) and Mass Spectrometry

The SPE eluate was subjected to reversed phase high performance liquid chromatography (RP-HPLC) using a SunFire Prep C_18_ (90 Å; 5 µm; 250 × 10 mm, Waters, Milford, MA, USA) or a reverse-phase column Jupiter C_5_ (300 Å, 10 µm, 250 ×10 mm, Phenomenex, Torrance, CA, USA) columns The eluate was separated using a linear gradient of 0–60% MeCN/0.05% TFA over 60 min, and a flow rate of 5 mL/min. Fractions were dried under vacuum and reconstituted in MQ–H_2_O before bioactivity testing. Active fractions were submitted for purity and mass measurements by mass spectrometry (MS).

Peptide purity and mass measurements were conducted using HPLC hyphenated with photodiode-array detection and electrospray ionization mass spectrometry (LC-PDA-ESI-MS). The system (Waters) consisted of an Alliance 2690 separation module connected to a 2998 photodiode array detector reading from 190 to 500 nm in 1.2 nm increments and a ZQ single quadrupole instrument, equipped with an electrospray LC interface. Samples were dissolved in water. The samples were separated using a Waters Sunfire C_18_ (5 µm, 2.1 × 100 mm) column. Linear gradients ran from 5 to 50% MeCN/0.05% TFA and a flow rate of 0.2 mL/min. MS analyses were performed in the positive ion mode with a cone voltage of 30 V. N_2_ was used as desolvation gas (flow of 1000 L/h) and cone gas (flow of 50 L/h). The data was recorded in the continuum mode of acquisition and the quadrupole scanned from *m/z* 100 to 2000. Ion signals were recorded using the MassLynx v4.1 (Micromass) software (Waters, Milford, MA, USA). The series of multiply charged protonated molecular ions acquired from the scan were used to calculate non-protonated average molecular masses.

High resolution mass spectrometry (HR-MS) was conducted using a 1290 Infinity UHPLC system hyphenated with a 6540 Q-TOF mass spectrometer with an ESI ion source, controlled by the MassHunter software (all Agilent, Santa Clara, CA, USA). Linear gradients went typically from 5 to 60% MeCN/0.1% Formic Acid (FA) with a flow-rate of 0.4 mL/min and column heated to 40 °C. Acquisition was in the positive ion mode at 2 GHz, drying gas eight L/min, Nebulizer gas 35 Psig, capillary voltage 3.5 kV, fragmentor 175 V, skimmer 65 V, *m/z* range varied with a maximum of 50–3200. Drying and nebulizer gas was N_2_ and the reference masses were 121.050873 and 922.009798 Da.

Molecular weight measurement was also carried out by matrix-assisted laser desorption ionization (MALDI), on an Ultraflex TOF-TOF (Bruker Daltonik, Bremen, Germany) instrument. The molecular mass was determined in linear positive ion mode, using samples prepared by the dried droplet method using as a matrix 2.5-dihydroxybenzoic acid (10 mg/mL in 70% acetonitrile with 0.1% TFA).

### 5.3. Sequencing and Structure Elucidation

Reduction was performed by dissolving ~20 nmol peptide in 100 µL 0.5 M Tris HCl/1 mM EDTA/6 M guanidine HCl and adding 5 µL 2.2 M dithiotreitol (DTT; Sigma-Aldrich, St. Louis, MO USA). The mixture was flushed with N_2_ to prevent oxidation and incubated for 16 h at 37 °C. The following alkylation was achieved by adding 5 µL 4-vinylpyridine (Sigma-Aldrich, St. Louis, MO USA) and incubating for 20 min at 37 °C. The reaction was stopped by RP-SPE, as previously described, eluting with 80% MeCN/0.05% TFA. Reduction and alkylation of Ueq 12-1 followed by Edman degradation sequencing were performed at Eurosequence (Groningen, The Netherlands). Protein sequence data are available in the UniProt Protein Database under the accession number C0HK26.

### 5.4. Precursor Determination and Gene Synthesis

Total RNA of *U. eques* was purified from the tentacles using the Trisol^®^ Reagent (Ambion, Burlington, ON, Canada) as described in the manufacturer’s protocols. RNA reverse transcription into cDNA was performed using the MINT kit (Evrogen, Moscow, Russia) according to the manufacturer’s recommendations. Rapid amplification of cDNA ends (3′-RACE) with the universal primer T7cap (GTA ATA CGA CTC ACT ATA GGG CAA GCA GTG GTA ACA ACG CAG AGT), and degenerated primers UE-d1 (TGC TAC CCA GGA CAR CCN GGN TG) and UE-d2 (GTC CGA AYT AYT GYG ARG GNG C) was carried out for 3′-terminus determination. 5′-terminus determination was performed by 5′-RACE using the universal primer T7cap, and the reverse primers UE-r1 (CAA GCA CAG CAG CAT CTA TCT) and UE-r2 (AGC TAA AGT GCA CTA GCC GCA). DNA sequencing was carried out using an ABI 3730 DNA Analyzer (Applied Biosystems, Carlsbad, CA, USA). The cDNA sequence of Ueq 12-1 was submitted to EMBL Nucleotide Sequence Database with submission number LT600337.

The DNA sequence encoding the peptide Ueq 12-1 was constructed from four synthetic oligonucleotides using the PCR technique. The primers UE-dir1 (GAA GAT CTA TGT GCT ATC CGG GCC AGC CGG GCT GCG GCC ATT GCT CCC GT) containing Met-codon for BrCN cleavage, UE-dir2 (GCC ATT GCT CCC GTC CGA ATT ATT GCG AAG GCG CGC GTT GCG AAT CCG GC), UE-rev1 (GAC TCG AGC TAC GCG CAG CAG CAA CGA TCG CCG GAC GCA TCG CAC CAA TG), and UE-rev2 (CGC ATC GCA CCA ATG ATC GGA GCC GCA ATC ATG AAA GCC GGA TTC GCA AC) were used for PCR fragment amplification. The target PCR fragment was gel-purified and cloned into the expression vector pET32b+ (Novagen, Madison, WI, U.S.A). The resulting construct was verified by sequencing using the ABI 3730 DNA analyzer. Nucleotide sequence data are available in the European Nucleotide Archive under the accession number LT600337.

### 5.5. Bioinformatics

The BLAST search engine [55], the Antimicrobial Peptide Database [56], the InterProScan tool [57] and StellaBase [58] was used to search for homologues sequences. The SignalP4.1 server [59] was used to predict the potential cleavage site of the signal peptide with the default setting for D-cutoff values and no TM regions selected. The theoretical molecular weight and the pI value of Ueq 12-1 were calculated using the Expasy program [60], while ChemCalc [61] was employed for theoretical monoisotopic mass elucidation.

### 5.6. Recombinant Peptide Production

The recombinant Ueq 12-1 was produced as a fusion protein with a thioredoxin domain using an *Escherichia coli* SHuffle^®^ T7 Express strain (New England Biolabs, Ipswich, MA, USA). The expression vector was transformed into competent cells, which were then cultivated at 37 °C in LB medium containing ampicillin at a concentration of 100 µg/mL. Expression was induced at a culture density of ~0.6–0.8 (OD_600_) by adding isopropyl-1-thio-β-d-galactopyranoside up to 0.2 mM. The cells were then cultivated for 18 h at 25 °C. After cultivation, the cells were harvested by centrifuging for 5 min at 6000× *g*, re-suspended in buffer for metal affinity chromatography (400 mM NaCl, 20 mM Tris-HCl, pH 7.5), ultrasonicated, and centrifuged for 15 min at 9000× *g* to remove all insoluble particles. The fusion protein was purified using the TALON Superflow metal affinity resin (Clontech, Mountain View, CA, USA) according to the manufacturer’s protocol. The fusion protein was diluted to 1 mg/mL and cleaved overnight with CNBr in the dark at room temperature, as previously described [62]. HCl was added to a final concentration of 0.2 M, and the CNBr molar ratio to peptide was adjusted to 600:1. In order to isolate the properly folded recombinant peptide, the reaction mixture was subjected to two separation steps: (1) RP-HPLC using a Jupiter C5 (250 × 10 mm, Phenomenex, Torrance, CA, USA) column and a linear gradient from 0% to 60% MeCN over 60 min (flow of 10 mL/min); and (2) RP-HPLC using a Vydac C18 (250 × 4.6 mm, Grace, Williamsburg, MI, USA) column and a linear gradient from 0% to 40% MeCN over 40 min (flow of 1 mL/min). The purity of the peptide was confirmed by MALDI-TOF MS and N-terminal sequencing.

### 5.7. NMR Spectroscopy and Calculation of Spatial Structure

The NMR sample was prepared by dissolving 0.8 mg of recombinant Ueq 12-1 in 350 µL buffer containing 5% D_2_O, 1 mM sodium azide, pH 3.2. All spectra were recorded at 303 K using a Bruker Avance III 600 MHz spectrometer (Bruker BioSpin, Billerica, MA, USA), equipped with triple resonance cryogenic probe. Proton, ^15^N and ^13^C resonance assignment was obtained via the standard procedure [63], based on the MLEV-TOCSY (80 ms mixing time), NOESY (40 and 80 ms mixing times), ^1^H, ^13^C-HSQC, ^1^H, ^15^N-HSQC and DQF-COSY spectra. After the set of NMR spectra was recorded, the sample was lyophilized and dissolved in pure D_2_O buffer as described above with the same contents to measure the rate of proton-deuterium exchange of amide groups, and additional NOESY (80 ms mixing time) and DQF-COSY were recorded.

Spatial structure calculation was performed using the simulated annealing/molecular dynamics protocol as implemented in the CYANA software package version 3.0 (L.A.Systems, Inc., Taito Tokyo, Japan) [29]. Upper interproton distance constraints were derived from the NOESY (τm = 80 ms) cross-peaks via a 1/*r^6^* calibration. Torsion angle restraints and stereospecific assignments were obtained from J couplings and NOE intensities. ^3^J_HNHα_ couplings were measured by the lineshape analysis of the cross-peaks in NOESY spectra and ^3^J_HαHβ_ coupling constants were obtained using the ACME software [64] in the DQF-COSY spectrum of Ueq 12-1 in D_2_O solution (relaxation delay 3 s). Hydrogen bonds were introduced based on the deuterium exchange rates of amide protons. Disulfide connections were determined in the course of the structure calculation.

Visual analysis of the calculated structures and figure drawings were made using the MOLMOL software (version number 2k2) [65]. The obtained spatial structure was validated and analyzed using the PDB-sum server [66]. Analysis of the 3D structure homology was performed by the PDBeFold analysis tool [37]. Potential grid was calculated using APDS software [67]. Chemical shifts, NMR constraints and derived atomic coordinates (10 models) of Ueq 12-1 were deposited into the Protein Data Bank [68], accession code 5LAH.

### 5.8. Antibacterial Activity Assay

Antibacterial activities were tested against a selection of Gram-positive and Gram-negative bacteria: *Corynebacterium glutamicum* (ATCC 13032), *Staphylococcus aureus* (ATCC 9144), *Pseudomonas aeruginosa* (ATCC 27853), and *Escherichia coli* (ATCC 2592). All isolates were grown at room temperature in Mueller Hinton Broth (MHB; Difco Laboratories, Detroit, MI, USA). Peptide concentration was determined by weighing and was diluted in water to a concentration of 200 µM and serial twofold dilutions were made before antibacterial activity testing. Antibacterial screening and minimal inhibitory concentration (MIC) determination were performed in 96-well microtitre plates, as previously described [69]. Briefly, 50 µL of test fractions were incubated with 50 µL of a suspension of an actively growing culture of bacteria diluted to a final concentration 1.3 − 1.5 × 10^4^ bacteria/mL. Bacterial growth was assayed every hour by measurement of the optical density (OD_595_) using Envision 2103 multilabel reader, controlled by the Wallac Envision manager (PerkinElmer, Waltham, MA, USA). The MIC value was defined as the lowest concentration of peptide resulting in no bacterial growth as determined by OD_595_ measurements. The purified peptide was tested in two parallels.

### 5.9. TRPA1 Activity Assays

The TRPA1 gene (AY496961.1) was amplified from rat brain, cloned into a pcDNA4TO vector and sequenced.

#### 5.9.1. Fluorescent Assay of Calcium Influx

The cell line CHO (Chinese Hamster Ovary), stably expressing rat TRPA1, was produced using the T-Rex System (Thermo Fisher Scientific Inc., Waltham, MA, USA) according to the manufacturers’ protocol. Shortly, a vector pcDNA4/TO with cDNA encoding rat TRPA1 was made, and transfected into CHO cells carrying the regulatory vector pcDNA6/TR (encoding the tetracycline repressor). The CHO cells were grown at 37 °C and 5% CO_2_ in DMEM/F12 (1:1) medium containing 10% fetal calf serum, After 2 weeks of selection by blasticidin (5 µg/mL) and zeocin (250 µg/mL), single colonies were screened using the agonist-induced [Ca^2+^] uptake assay. TRPA1 expression was induced by adding up to 1 µg/mL tetracycline 24 h before testing. Positive clones were used for experimentations. Fluorescent assays were performed using a tablet spectrophotometer with integrated automatic liquid dosing system NOVOstar (BMG LABTECH, Ortenberg, Germany). rTRPA1-CHO cells were seeded into black-walled clear-bottomed 96-well plates at a density of 75,000 cells per well and cultured overnight at 37 °C (complete media containing 1 µg/mL of tetracycline). TRPA1-expressing cells were stained with the calcium indicator Fluo-4AM using the Fluo-4 Direct™ Calcium Assay Kit (Thermo Fisher Scientific Inc., Waltham, MA, USA), and incubated in the dark successively at 37 °C and 25 °C for 60 min each time. The control (buffer alone) and serial dilutions of Ueq 12-1 (concentrations ranging from 0.3 to 750 µM) were added to the cells and measurements were immediately carried out. Fluorescent signals were monitored before and after the addition of the TRPA1 agonist. The measurements were performed at room temperature and pH 7.4.

#### 5.9.2. Electrophysiology

cRNA of TRPA1 (AY496961.1) was synthesized from a NarI-linearized pVAX1/TRPA1 plasmid using a HiScribe™ T7 High Yield RNA Synthesis Kit (New England Biolabs, Ipswich, MA, USA) according to the manufacturers’ protocol for capped transcripts. The cRNA transcripts (2–5 ng) were injected in *Xenopus laevis* oocytes. The injected oocytes were incubated for 2–7 days at 15–19 °C in sterile ND-96 medium containing 100 mM NaCl, 2.5 mM KCl, 1.8 mM CaCl_2_, 1 mM MgCl_2_, 5 mM 4-(2-hydroxyethyl)-1-piperazineethanesulfonic acid HEPES, pH 7.4, supplemented with 50 µg/mL gentamycin.

Electrophysiological measurements were carried out in a bath perfused with Ca^2+^-free solution that contained 100 mM NaCl, 2.5 mM KCl, 1 mM MgCl_2_, 5 mM HEPES, pH 7.4. Oocytes were impaled with two glass microelectrodes filled with 3 M KCl and connected to a GeneClamp 500 amplifier (Axon Instruments, Union City, CA, USA). Oocytes were clamped at −20 mV, recording of inward/outward currents made at repeated step to −100 mV for 80 ms following voltage ramp from −100 mV to +100 mV for 200 ms every 4 s. Diclofenac (300 µM in Ca^2+^-free solution) [70] or Allyl isothiocyanate (AITC, 100 µM in Ca^2+^-free solution) [71] were used to activate the channel. Experiments were performed at room temperature (22–24 °C). The data were filtered at 20 Hz and digitized at 100 Hz by an AD converter L780 (LCard, Moscow, Russia) using in-house software.

### 5.10. In Vivo Experiments

#### 5.10.1. Animal Models

All experiments were approved by the Animal Care and Use Committee of the Branch of the IBCh RAS (Pushchino, Russia) protocol number No. 492/16. Adult male CD-1 mice (weight 20–30 g; obtained from the Animal Breeding Facility Branch of Shemyakin-Ovchinnikov Institute of Bioorganic Chemistry, Russian Academy of Sciences, Pushchino, Russia Federation) were housed at room temperature (23 ± 2 °C) and subjected to a 12 h light–dark cycle with food and water available *ad libitum*. Ueq 12-1 was dissolved in saline. The significance of the data was determined by Analysis of variance (ANOVA) followed by Turkey’s post-hoc test. Data are presented as mean ± SEM. No explicit power analysis was performed prior to the experiments to determine sample size, since we had no means to reliably estimate the size and variability of the effects of the peptide on mice. Since highly significant results were obtained from this set of experiments, no further animals were sacrificed.

#### 5.10.2. Test on Pain/Thermal Hyperalgesia-Evoking Activity

Ueq 12-1 (2.5 µg/10 µL) or saline (10 µL) were injected into the left hind-paw. Licking and guarding behavior was monitored for 15 min after injection. Withdrawal latency of injected hind-paw from a hot plate (53 °C) was observed 2 h after injection.

#### 5.10.3. “Open Field” Test on Locomotor Activity

The effects of Ueq 12-1 on spontaneous locomotor activity of mice were assessed using TSE Multi Conditioning System Extended Advanced with TSE ActiMot (Activity & Hole Board Measuring System) module and test arena “Open field” (TSE Systems, Inc. Chesterfield, MO, USA). Locomotor activity was recorded for 15 min. Peptide (0.2 mg/kg) and saline were administered intravenously (i.v.) 30 min before testing.

#### 5.10.4. Allyl Isothiocyanate (AITC)-Induced Nocifensive Beaviour

The TRPA1 agonist AITC was used to activate peripheral TRPA1 receptors and induce nocifensive behaviors in mice [72]. To produce nocifensive behavioral response AITC (20 µL, 0.5% in saline) was injected into the plantar surface of the hind-paw. Ueq 12-1 (0.2 mg/kg) or saline were administrated i.v. 30 min before AITC administration. The duration of paw guarding and number of licks were recorded for 5 min after injection of AITC. The diameter of the paw was evaluated before the test and 2, 4 and 24 h after injection using an electronic digital caliper.

#### 5.10.5. Complete Freund’s Adjuvant (CFA)-Induced Inflammation and Thermal Hyperalgesia

Paw inflammation and thermal hyperalgesia was induced using Complete Freund’s Adjuvant (CFA) suspended in an oil/saline (1:1) emulsion. The mice were injected with 20 µL of CFA emulsion into the plantar surface of the left hind-paw. The control mice were injected with 20 µL of saline. After 24 h, Ueq 12-1 (0.2 mg/kg and 1 mg/kg) or saline were i.v. administrated. Paw withdrawal latency was recorded using a hot plate (53 °C) 30 min after peptide or saline administration. The diameter of the paw was evaluated before CFA injection, before Ueq 12-1 or saline administration and 2, 4, 24 h after Ueq 12-1 or saline administration using electronic digital caliper. Percent inflammation was calculated according to the formula ((Postdose paw diameter − Naive paw diameter)/(Predose paw diameter − Naive paw diameter)) × 100.

## Figures and Tables

**Table 1:** Statistics for the best Ueq 12-1 structures and NMR input data.

**Distance and Angle Restraints**	
Total NOEs	308
intraresidual	88
interresidual	220
sequential (|i − j| = 1)	104
Medium range (1 < |i − j| ≤ 4)	36
Long range (|i − j| > 4)	80
Hydrogen bond restraints (upper/lower)	56/56
S-S bond restraints (upper/lower)	15/15
J-couplings	70
J_HNHα_	34
J_HαHβ_	36
Total restraints/per residue:	523/12
**Statistics of calculated set of structures**	
CYANA target function (Å^2^)	1.77 ± 0.04
Restraint violations	
distance (>0.2 Å)	3
angle (>5°)	0
**RMSD (Å) Elements with defined structure (6–27)**	
Backbone	0.38 ± 0.15
All heavy atoms	1.03 ± 0.14
**Ramachandran analysis**	
% residues in most favored regions	75.8
% residues in additional allowed regions	21.2
% residues in generously allowed regions	3.0
% residues in disallowed regions	0.0

**Table 2:** PDBeFold structure alignment results of Ueq 12-1 (PDB ID: 5LAH). The Q-score is calculated as Q = Nalgn × Nalgn/((1 + (RMSD/R_0_)^2^) × Nres1 × Nres2) where R_0_ = 3 Å, The Z-score measures the statistical significance of a match. The higher Z-score, the higher statistical significance of the match.

Peptides	PDB Code	N res	N Align	% Iden	Score		RMSD
					Q	Z	
Cryptdin-4, alpha-defensin from mouse, a-defenM4, Crp4	1tv0	32	29	21	0.46	4.2	1.54
Revised solution structure of platypus DLP-2	1zue	42	38	13	0.40	4.0	2.85
Human defensin HBD-2	1e4q	37	34	18	0.39	3.7	2.67
DLP-4 from platypus	1zuf	42	38	13	0.38	3.1	3.00
Human defensin HBD-1	1e4s	36	31	19	0.38	5.5	2.24
Kalata B12 from *Oldenlandia affinis*	2kvx	28	22	14	0.31	2.8	1.42
Anthopleurin-A from the sea anemone *Anthopleura xanthogrammica*	1ahl	49	30	23	0.26	5.3	2.27
CGNA toxin from the sea anemone *Condylactis gigantea*	2h9x	47	30	20	0.25	5.0	2.50
